# PurificationDB: database of purification conditions for proteins

**DOI:** 10.1093/database/baad016

**Published:** 2023-04-03

**Authors:** Olivia Garland, Mariia Radaeva, Mohit Pandey, Artem Cherkasov, Nada Lallous

**Affiliations:** Vancouver Prostate Centre, University of British Columbia, 2660 Oak Street, Vancouver, BC V6H 3Z6, Canada; Vancouver Prostate Centre, University of British Columbia, 2660 Oak Street, Vancouver, BC V6H 3Z6, Canada; Vancouver Prostate Centre, University of British Columbia, 2660 Oak Street, Vancouver, BC V6H 3Z6, Canada; Vancouver Prostate Centre, University of British Columbia, 2660 Oak Street, Vancouver, BC V6H 3Z6, Canada; Vancouver Prostate Centre, University of British Columbia, 2660 Oak Street, Vancouver, BC V6H 3Z6, Canada

## Abstract

The isolation of proteins of interest from cell lysates is an integral step to study protein structure and function. Liquid chromatography is a technique commonly used for protein purification, where the separation is performed by exploiting the differences in physical and chemical characteristics of proteins. The complex nature of proteins requires researchers to carefully choose buffers that maintain stability and activity of the protein while also allowing for appropriate interaction with chromatography columns. To choose the proper buffer, biochemists often search for reports of successful purification in the literature; however, they often encounter roadblocks such as lack of accessibility to journals, non-exhaustive specification of components and unfamiliar naming conventions. To overcome such issues, we present PurificationDB (https://purificationdatabase.herokuapp.com/), an open-access and user-friendly knowledge base that contains 4732 curated and standardized entries of protein purification conditions. Buffer specifications were derived from the literature using named-entity recognition techniques developed using common nomenclature provided by protein biochemists. PurificationDB also incorporates information associated with well-known protein databases: Protein Data Bank and UniProt. PurificationDB facilitates easy access to data on protein purification techniques and contributes to the growing effort of creating open resources that organize experimental conditions and data for improved access and analysis.

**Database URL** https://purificationdatabase.herokuapp.com/

## Introduction

Protein isolation and purification is a critical step for studying proteins in the fields of biochemistry, drug discovery and structural biology (1). A successful purification process enables separation of protein of interest from a complex cellular mixture, containing other proteins, chaperones, nucleic acids, polysaccharides and lipids. The complex nature of the protein purification process emphasizes the importance of monitoring and maintaining the quality of proteins. There exists a broad range of separation methods to purify proteins using liquid chromatography such as affinity, size exclusion, and hydrophobic, among others.

Liquid chromatography methods can be applied for both analytical and/or preparative goals. In analytical applications, relative proportions of analytes in a mixture can be measured using column chromatography (2). As a preparatory technique, it allows for the separation of components in a mixture for further use. This could include the removal of contaminates or conditioning samples before additional purification techniques (3). For example, an important early step in the protein isolation procedure is the removal of proteases by chromatography to avoid proteolysis in downstream steps, affecting the quality (4). Another common use of column chromatography includes protein purification prior to crystallization for the elucidation of three-dimensional structure. Importantly, the high cost of crystallography requires that the protein samples are prepared at high purity and yield (5). As such, to achieve reproducibility of results and avoid accidental crystallization of contaminant proteins, purification procedures used for crystallization are calibrated precisely.

Liquid chromatography involves the application of a mixture of molecules, known as the ‘mobile phase’, to a surface known as the ‘stationary phase’. The stationary phase dictates which molecules can pass through the column and at which speed they do so based on their physical and chemical interactions with the stationary phase. Molecules are eluted from the stationary phase by competition or by changing their ionic strength through alteration of pH or the addition of salt solution (6). The most commonly used chromatography techniques are size exclusion chromatography (SEC), ion exchange chromatography (IEC), hydrophobic interaction chromatography, and affinity chromatography.

The SEC separation is achieved by passing the mobile phase through a packed resin containing pores of different sizes (7). Molecules are partitioned between these phases as a function of their relative sizes. Molecules smaller than the pore size of the resin will remain part of the internal volume of the chromatography column, while molecules that are much larger than the pore size will elute quickly from the column (3).

Another commonly applied chromatography technique for protein purification is IEC. The separation principles of IEC are based on electrostatic attractions between charged particles on the surface of the stationary phase and charged areas of the molecules in the mobile phase. The charges of the molecules in the mobile phase and on the resin are balanced by counterions present in salt or buffer ions. When molecules bind to the resin, these counterions are displaced mediating so-called ‘ion exchange’. Molecules are eluted from the resin by increasing ionic strength of the buffer (8).

Affinity chromatography is another method of protein separation that utilizes a protein’s characteristic affinity for specific chemical groups as the principle for separation. Protein affinity tags, which are peptide sequences, are genetically engineered to the N- or C-terminus of the protein of interest. The stationary phase is designed to have functional groups which make a complex with these affinity tags when the mobile phase is passed through the column (6). One of the most common tags used is polyhistidine tag, which is used in immobilized metal affinity chromatography, where the tag has high affinity for metal ions on the resin (9). Other examples of affinity tags include glutathione S-transferase, which has high affinity for glutathione and can be coupled to a Sepharose matrix, and maltose-binding protein, which has natural affinity for α-(1–4)maltodextrin and also improves the solubility of the protein of interest (10, 11).

Although SEC, IEC and affinity chromatography are the focus of this work, there exist other specialized chromatography techniques worth mentioning. Some examples include biorecognition chromatography, which is a type of affinity chromatography based on molecular recognition (12), and similarly immunoaffinity chromatography, which uses antibodies in the stationary phase to act as a biologically related binding agent (13). Another example is hydrophobic interaction chromatography, which exploits differences in hydrophobicity between stationary and mobile phase molecules (14). There also exist mixed-mode chromatography methods, in which the stationary phase interacts with solute by more than one mechanism (15).

In addition to choosing a separation technique, researchers must also consider the composition of buffer solutions based on the application and the characteristics of the protein. In liquid chromatography, buffers are used in several steps of the protocol: as part of the mobile phase and then as an eluent to remove molecules that are interacting with the stationary phase. The general role of buffering agents is to resist changes in pH and maintain protein stability. However, buffers also serve additional roles, for example, in IEC, the elution step requires a higher salt concentration relative to that of the buffer used in the mobile phase, as the salt must compete with the bound proteins to remove them from the resin (8). Another characteristic of the buffer which must be tuned correctly is the pH. Using a pH outside of a protein’s optimal range can cause unfolding, aggregation and loss of functional activity, ultimately reducing the purity and activity of the protein of interest (16). There are also occasional additives to buffer solutions that play other roles, for instance, detergents improve protein solubility and prevent denaturation (17).

To successfully purify a protein of interest, one needs to calibrate a set of conditions and decide on the components and concentrations of buffer solution. With the abundance of such parameters, it is challenging to estimate the right settings *a priori*. Therefore, biochemists need to consider protein size, type and location of the tag, isoelectric point, presence of disordered regions, solubility of the protein, etc. They often search the literature for reports of successful purification of their protein of interest. Unfortunately, there are many roadblocks to deriving relevant information such as lack of accessibility to journals and their supplementary information and lack of detailed experimental methods in published works. Furthermore, some articles do not fully describe the procedures performed but rather reference other articles, so finding a detailed protocol may require cumbersome surfing through a series of works. Additionally, authors might use different units of measurements and nomenclature conventions, thereby further complicating the retrieval and comparison of various purification protocols. Thus, there is a need for an easily accessible, comprehensive and standardized source of information for protein purification conditions. To this end, we developed PurificationDB, an open-access database that contains curated and focused information on buffer conditions for use in SEC, IEC and affinity chromatography for protein purification.

PurificationDB is a structured knowledge base, developed by automating information extraction from thousands of relevant published articles. We use domain knowledge to write rules for named-entity recognition. Our database contains relevant buffer conditions including pH, concentrations of salts, buffering agents, detergents and other additives. We have also included connections to related databases by UniProt identifiers (UniProt ID) (18), Protein Data Bank (PDB) identifiers (PDB ID) (19) and sequence information. With such information, we implemented features that allow the user to search by protein name, UniProt ID, PDB ID or sequence to easily find the information they require in our database. Our web interface can be found at purificationdatabase.herokuapp.com.

## Methods

### Collection of literature

To collect information on protein purification buffer conditions, we fetched the information from papers linked to one or multiple protein crystal structures deposited in PDB (19). To do so, we first retrieved all available PDB IDs of protein structures belonging to *Homo sapiens* and having the label ‘protein only’ (i.e. protein–nucleic acid complexes were not considered). The PDB codes were then used to retrieve digital object identifiers (DOIs) of the corresponding papers. This yielded 16 204 unique DOIs. Subsequently, we developed a visual automation script using SikuliX to download full texts of these studies (20). For a handful of cases where our automation workflow failed, we manually downloaded the respective papers using our institutional authorization.

### Data curation and wrangling

The first step for data curation from this large set of unstructured data was the extraction of text from the PDF documents using a Python library, PDFMiner (21). Once the texts were extracted from the file, the Regular Expression library in Python was utilized for text cleaning and preprocessing. This included the removal of all non-ASCII (anything that is not English characters, punctuations, numbers 0–9 or dashes) characters. All special characters were removed except the full stop and percentage symbol as they are relevant to specifying the concentration values. This improved the robustness of our text searching algorithms as it standardized the different representations of the text.

To construct the knowledge base, we designed a named-entity recognition algorithm based on nomenclature of chromatography steps and buffer conditions and components. In particular, the algorithm searches for sentences that contain words specified in a reference table and extracts information from these sentences. The reference tables contained words determined using expert biochemist knowledge and literature. One such reference table contained common terms, synonyms and acronyms for chromatography techniques, for example, ‘size-exclusion chromatography’, ‘SEC’ and ‘gel-filtration chromatography’. Additionally, we added manufacturer names as they are often used to refer to chromatography techniques, for example, ‘Superdex’ is the name of the SEC column manufactured by Cytiva and Sigma-Aldrich (22, 23). We also developed reference tables of common names and synonyms of chemicals relevant to chromatography. For instance, sodium chloride is a commonly used salt in the buffer for SEC, so our reference table contains synonyms and various chemical formula representations of sodium chloride. Reference tables were not only used for search algorithms in the retrieval process, but were also used to develop a classification system to organize queried entries from the standardized database and present them to users.

To reduce the search space, we split the text into sentences and searched for keywords from the chromatography reference tables. If a sentence contained a keyword, the sentence in which it appears and the ones before and after were considered in the next step of the search. We found that this procedure improved the precision of the search algorithm and ensured that irrelevant information (e.g. crystallization buffer conditions) are not included. Additionally, the index of a sentence with a keyword is saved to later infer the order of chromatography steps.

After the search space reduction, the next step was to search for experimental conditions within the retrieved sentences. Rules were defined based on linguistic patterns from the literature of this domain as we observed that the language used to describe experimental protocols tends to be quite standard. One example of such a pattern is where the measurement and its unit, if applicable, were placed relative to their respective characteristic or buffer component. The simplest example is the pH value that almost always comes after the word ‘pH’. The chemical component reference table enabled searching for relevant buffer names and concentration values. Importantly, the retrieved concentration values were then standardized so that the resultant database contains easily comparable values.

After performing feature extraction of buffer conditions from papers, we connected these conditions to their associated proteins in our knowledge base. To do this, the associated PDB IDs were mapped to the corresponding UniProt IDs using the UniProt database identifier mapping tool (18, 24). Once UniProt IDs were found, we then used UniProt API to retrieve the protein name and its synonyms, as well as sequences corresponding to each of its associated PDBs. Importantly, the protein sequence corresponds to the portion included in the PDB structure rather than the whole protein sequence. This might be important when different domains of the protein require slightly different buffer conditions. The metadata including protein sequence, name and UniProt ID were then used in the database’s search tools as discussed in the next section.

### Database and website implementation

The website interface for accessing the purification database was designed using React for the front end and Node environment for the back-end (25). Data were stored using MySQL database and is queried using MySQL commands (26). The website and database are hosted using Heroku, a cloud application platform. We implemented function which allows the user to search by protein name, UniProt ID or sequence.

## Results

### Dataset statistics

Of the original 16 204 fetched paper DOIs, ∼11 700 full texts were successfully extracted. Of those, 4732 papers contained relevant buffer conditions as determined by our search algorithm. The buffer conditions were first classified based on the type of chromatography they were used for. There is a notable difference between the number of buffer conditions for SEC versus for IEC and affinity chromatography (Table 1).

**Table 1.** Table of number of database entries corresponding to each chromatography type

Chromatography type	Number of entries
Size exclusion	4650
Ion exchange	261
Affinity	344

Upon manual investigation of several hundred such examples, we observed that a striking number of papers reported the type of the purification method but did not report the purification buffers. These papers only specify a single buffer used for the final chromatography step. SEC is often the last step of purification before crystallization or functional assays, which is why we see such a big difference between the number of buffer conditions extracted for SEC relative to IEC and affinity (Table 1).

To give the user a better understanding of subsequent experimental steps the buffer was used in, and to determine if it is used as the preparation or elution buffer, we include the portion of the text where the buffer is described in our database.

The extracted buffer conditions were then classified based on chemical components and their role in the buffer, i.e. salt, buffering agent, detergent or additive (Table 2). The variety of buffer components that we found reflects the complexity of purification conditions as different proteins might require specific additives for successful purification. In general, buffering agents are used to adjust and stabilize the pH of a solution. Thus, all the retrieved buffers contain a salt component (primarily sodium chloride). Salt ions act as buffers by binding H+ ions present in the solution and thus resisting the change in the pH (16). We also found a variety of other components such as detergents that improve protein solubilization and prevent denaturation (17). However, only 15% of reported buffers contain a detergent component highlighting that not all proteins suffer from low solubility in aqueous solutions. Around a third of reported buffers contain dithiothreitol (DTT) which plays a role in the stabilization of proteins mainly by preventing oxidation of thiol groups of cysteine residues maintaining them in reduced states (27). Other additives that were found only in a very small fraction of papers (<1%) serve as supplementary agents. For instance, sodium azide (found in 0.5% of papers) is used to prevent bacterial contamination (28).

**Table 2.** Values available in PurificationDB database and their associated densities

Classification	Component	Number of papers	% Density
Salt	Sodium chloride	4732	100
	Potassium chloride	60	1.27
	Calcium chloride	6	0.13
	Magnesium chloride	4	0.08
	Sodium acetate	26	0.55
	Disodium phosphate	51	1.08
	Monopotassium phosphate	25	0.53
	Monosodium phosphate	34	0.72
Buffering agent	Tris(hydroxymethyl)aminomethane (Tris)	2417	51.08
	Imidazole	121	2.56
	2-[4-(2-Hydroxyethyl)piperazin-1-yl]ethane-1-sulfonic acid (HEPES)	1456	30.77
	2-Ethanesulfonic acid	94	1.99
	Phosphate-buffered saline	24	0.51
	1,4-Piperazinediethanesulfonic acid (PIPES)	2	0.04
	Bis-Tris	16	0.34
Detergent	3-[(3-cholamidopropyl)dimethylammonio]-1-propaneulfonate (CHAPS)	17	0.36
Polyol	Polyethylene glycol	13	0.27
	Ethylene glycol	7	0.15
	Glycerol	747	15.79
Chaotrope	Urea	13	0.27
Reducing agent	Tris(2-carboxyethyl)phosphine	624	13.19
	2-Mercaptoethanol	31	0.66
	Dithiothreitol (DTT)	1461	30.87
Additive	Egtazic acid (EGTA)	38	0.8
	Ethylenediaminetetraacetic acid (EDTA)	432	9.13
	Guanosine diphosphate (GDP)	17	0.36
	Sodium azide	27	0.57
	Sodium glutamate	1	0.02
	Adenosine triphosphate (ATP)	28	0.59
	Methionine	2	0.04
	Citric acid	2	0.04

All the protocols contained information about pH as this parameter ensures that the protein maintains its activity and stability during the purification process. The purity and quality of the protein can be drastically undermined at suboptimal pH. Each protein has a range of physiological pH that is determined by amino acid composition and function. Proteins can withstand fluctuations in pH as they move between subcellular compartments, which have different pH values, to carry out their biological functions (29). This explains why some of the proteins purified in multiple experiments have slightly different reported pH values. If affinity tags are used for purification, it is also important to consider the pH at which they function. Depending on the type of chromatography deployed, biochemists generally use the general rule that the pH of the buffer solution should be away by 1.0 pH unit of the protein’s isoelectric point to avoid aggregation and precipitation and ultimately an unsuccessful purification (30).

**Figure 1.**
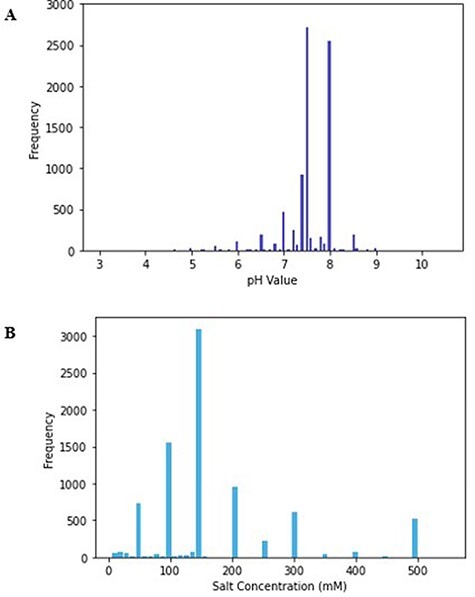
(A) Histogram of pH values in PurificationDB. (B) Histogram of sodium chloride salt concentration values in PurificationDB.

The minimum value of pH in the database is 3, while the maximum is 10.5, and the mode of the pH values was 7.5 (Figure 1). The mode reflects the physiological pH at which most proteins exist, pH 7.4. Even within a single cell, the pH generally ranges between 4.5 and 7.4; thus, proteins that reside in different subcellular compartments might have different optimal pH values (31).

We examined the minimum and maximum pH values from the database and found that they belong to proteins that naturally exist in extreme pH environments. For instance, a buffer with pH of 3 was used for SEC applied to the envelope glycoprotein, gp160, of human immunodeficiency virus 1 (PDB: 4JKP, UniProt: Q0ED31) (32). Gp160 contributes to virus transmission through contact with CD4 protein receptor on host T-cell membranes, which initiates the binding and subsequent introduction of the viral RNA. The virus is often transmitted during sexual intercourse where an acidic mucosa pool exists. Therefore, gp160, which naturally maintains its function in acidic conditions, was purified in a buffer with low pH as suggested from the parsed literature (33). On the other end, we found protein NaChBac (PDB: 6VWX, 6VX3, UniProt: Q9KCR8) that was purified using SEC in highly basic conditions with pH of 10 (34). The authors of this work noted that the protein was most compatible with several solubilizing detergents only at pH of 10. This work highlights that sometimes, pH values have to be altered away from natural protein’s pH range to accommodate other additives. An example of a protein that uses a buffer with a pH closer to neutral, i.e. 7.5, is that of *Mycobacterium tuberculosis* peptide deformylase (PDB: 3E3U, UniProt: P9WIJ3) (35). Peptide deformylase contributes to protein maturation by performing deformylation of the *N-*formylmethionine of newly synthesized polypeptides (35). This reaction takes place on the ribosome surface that has a typical pH environment of around 7.5 (36).

All the database entries contain a specification of a salt component of the buffer. Salt is an important component in buffer solutions as it influences the selectivity of protein interactions with the resin and the structural stability of the protein (37). Salts have the ability to screen Coulombic effects, which otherwise promote repulsions and consequent destabilization of the protein (38). This change in stability and solubility ultimately can lead to the Hofmeister series, i.e. precipitation of proteins, which happens via changes in the interactions between solvent and protein through preferential hydration (38). For these reasons, it is important to choose a salt concentration appropriate for the protein of interest. The physiological salt concentration ranges between 100 and 300 mM and over 70% of entries in PurificationDB fall into this range (39). In our database, the salt concentration ranges from 5  to 550 mM and has a mode of 150 mM (Figure 1B).

In addition, we mapped the PDB IDs of the proteins from the database to their respective UniProt IDs. Out of 4732 entries in the database, 3877 belong to unique UniProt IDs representing as many unique proteins. We also observed that 239 entries could not be mapped to a UniProt ID, and these mostly represented artificially constructed proteins. Furthermore, we observed that the protein set present in the PurificationDB is very diverse as the proteins belong to 3662 protein clusters defined by UniRef50 sequence clustering (40). We also retrieved corresponding protein sequence of each database entry by taking the start and end positions of PDB protein and selecting corresponding segments from whole sequences deposited in UniProt. This was done to ensure that the sequence of interest was preserved in its original form and does not include potential tags, mislabeled or missing residues, mutations and other issues that might present in PDB sequences.

Besides, we examined the proportion of proteins that were crystallized as one component compared to protein complex structures (Table 3). We found that the majority of entries in the database (66.7%) belong to buffers used for one component purification, while only a small fraction (8.9%) contained more than three proteins. Such distribution reflects the fact that most of the PDB entries represent single proteins rather than protein complexes. For multicomponent preparation, the buffer conditions need to be optimized to accommodate for the optimal activity and stability of all components and the interactions between them. Thus, buffers used to purify a single protein might differ from those used for purification of the same protein in complexes with partners. Since proteins that form complexes to perform their function would all exist within the same environment, we expect the pH of the purification buffer to reflect the pH of the environment. For example, one entry of the database corresponds to the human nuclear pore complex (PDB: 5A9Q), which maps to 10 UniProt entries (UniProt: O75694, P52948, P55735, P57740, Q12769, Q8NFH3, Q8NFH4, Q8WUM0, Q96EE3 and Q9BW27) and therefore 10 different proteins. This complex that is responsible for nucleocytoplasmic exchange is located on the nuclear membrane and exposed to the nucleoplasm and cytoplasm that have a pH of ∼7.3 in HeLa cells (41, 42). This reflects the buffer used in the parsed work to purify the protein complex from HeLa cells, which has a pH of 7.5 (43).

**Table 3.** Table of number of database entries corresponding to number of protein components associated with an entry

Number of components	Number of entries	% Density
1	2995	66.7
2	1096	24.4
>2	402	8.9

Entries with two or components indicate it is a complex

In addition to protein–protein interactions, we also investigated the number of entries which contained a protein with post-translational modifications (PTMs) (Table 4). This information was obtained through metadata associated with UniProt. We found 30.1% of entries in the database corresponded to entries with no PTMs, while a similar proportion, 28.1%, had one PTM.

**Table 4.** Table of number of database entries corresponding to number of PTMs (where information was available)

Number of PTMs	Number of entries	% Density
0	1425	30.1
1	1332	28.1
2	778	16.4
>2	958	20.2

### Website interface

**Figure 2.**
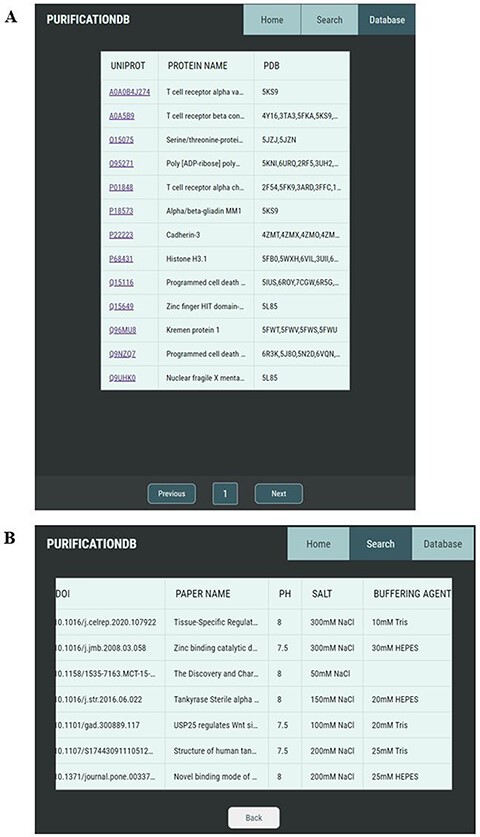
Pages of PurificationDB. (A) On the ‘Database’ page, users can browse entries of the database, labeled with their name, UniProt ID, and associated PDB IDs. (B) Upon selection of a UniProt entry O95271, entries from PurificationDB corresponding to this protein are displayed. In this case, we can see that there are several sets of buffer conditions published.

Our user-friendly interface allows for simple browsing and exploration of the protein purification conditions collected from the literature. The ‘Database’ tab allows the user to look through all entries of the database organized by UniProt ID (Figure 2A). Upon selection of a UniProt ID of interest, the user is brought to a new page corresponding to this entry (Figure 2B), which contains all unique corresponding entries, as several reported purification conditions could exist per one UniProt ID.

The ‘Search’ tab allows for the user to easily search the entire database by protein name, UniProt ID or sequence. As there are often many names which identify one protein, we developed a reference table which contains all UniProt protein names and associated alternative names for entries in our database. Therefore, upon user entry for the protein name search, this reference table is used to allow for a more robust search of proteins in our database to find the right entry. The protein name search is performed using MySQL’s SOUNDEX function, which converts a string to a code based on how the string sounds when spoken in English and can therefore be used to evaluate the similarity of two strings (44). This function yields similar entries to what the user has input as their search term.

## Conclusions

PurificationDB is a curated database with 4732 entries of buffer conditions for protein purification using SEC, IEC and affinity chromatography derived from the literature. The derived buffers were used in the preparation procedures for crystallization that yielded protein structures deposited in PDB (19). Thus, we reason that these buffer conditions are proven to be reliable and suitable for a particular protein of interest. For each entry in the database, we specify the type of the chromatography used and the conditions including pH, concentrations of salts, buffering agents, detergents and other additives. Importantly, the database uses standard terms for all the components allowing users to easily compare the conditions without expert knowledge of all the possible terms and acronyms. Additionally, we standardize the units of the concentrations to make the information more accessible. The use of common terms and standard units is important for future potential meta-analyses of the data.

Furthermore, the database entries are mapped to their corresponding PDB IDs and UniProt IDs along with protein sequence. We retrieved buffer conditions used to purify 3877 unique proteins that represent a broad range of sequence diversity. With this number, we estimate that we capture around 18% of all human protein assuming 21 306 unique proteins in the human proteome (45). As we retrieved the purification conditions from crystallization reports only, we acknowledge that the database might lack information on purification conditions for proteins that are challenging to crystallize, e.g. disordered proteins.

PurificationDB is hosted on a user-friendly web interface which allows users to search for entries to easily access conditions for their protein of interest. We developed this database to make information more accessible to researchers, so they no longer have to search for and read through multiple articles to find the experimental protocols for protein purification of their interest. This contributes to the growing effort of creating resources that organize experimental conditions and data for improved access and analysis. Other examples of such resources include LLPSDB, a database containing a collection of proteins involved in liquid–liquid phase separation with corresponding experimental conditions *in vitro* from the published literature, and PINT, a database of thermodynamic parameters and experimental conditions for protein–protein interactions (46, 47). PurificationDB represents another example of a knowledge base that facilitates easy access to data on protein preparation techniques.

All entries of buffer conditions that are part of PurificationDB are those that have led to successful purification and ultimately crystallization of proteins of interest as they are derived from entries in PDB. As such, data from PurificationDB may serve as a reliable training dataset for machine learning models or other methods for the prediction of conditions at which new proteins, which do not have published protocols associated with successful separation, may be purified.

In future, we hope to expand the ability of our data wrangling processes to collect experimental conditions not limited to a specified vocabulary and language rules for a more robust, through more sophisticated natural language processing techniques. Such development would expand the list of compounds discovered and could ultimately be applied to a collection of other types of experimental protocols.

## Data Availability

All database entries are freely available through our deployeed app at https://purificationdatabase.herokuapp.com/.
